# Effects of Hydroxytyrosol Against Doxorubicin-Induced Hepatotoxicity Through Alterations in Dardarin and Podocalyxin Expression in Rats

**DOI:** 10.7759/cureus.109870

**Published:** 2026-05-29

**Authors:** Elif Onat, Nevin Kocaman, Erkan Yilmaz, Serhat Hançer

**Affiliations:** 1 Department of Pharmacology, Adıyaman University, Adıyaman, TUR; 2 Department of Histology and Embryology, Firat University, Elazığ, TUR; 3 Department of Pharmacognosy, Adıyaman University, Adıyaman, TUR; 4 Department of Histology and Embryology, Gaziantep City Hospital, Gaziantep, TUR

**Keywords:** dardarin, doxorubucin, hydroxytyrosol, liver, podocalyxin

## Abstract

Introduction: This study investigated whether dardarin (LRRK2) and podocalyxin (PODX) contribute to the hepatoprotective effects of hydroxytyrosol (HT) against doxorubicin (DOX)-induced liver injury in rats.

Methods: Twenty-eight female Sprague Dawley rats were randomly assigned to four groups: Control, HT, DOX, and DOX+HT. DOX (10 mg/kg) was administered intraperitoneally as a single dose on day 1. HT was given orally at 100 mg/kg/day for 28 days. In the DOX+HT group, animals received DOX on day 1 followed by daily oral HT for 28 days. At the end of the experiment, liver tissues were collected for histopathological evaluation, and all findings were statistically analyzed.

Results: Histopathological assessments showed significantly elevated LRRK2 and PODX expression levels in the DOX group, while co-treatment with HT markedly reduced these increases (p<0.001). Additionally, DOX-induced hepatic fibrosis, leukocyte infiltration, congestion, and sinusoidal dilatation were substantially alleviated in the DOX+HT group (p<0.001).

Conclusion: HT demonstrated beneficial effects on histopathological and immunohistochemical changes in DOX-induced liver injury. The reduction in LRRK2 and PODX expression implies that these molecules may be involved in the mechanisms underlying HT-mediated hepatoprotection. These findings highlight the potential therapeutic value of HT in mitigating DOX-associated hepatotoxicity.

## Introduction

Doxorubicin (DOX), a potent anthracycline antibiotic, is widely used in the treatment of various malignancies, including breast, ovarian, and hematological cancers. However, its clinical use is limited by dose-dependent toxicities, particularly hepatotoxicity and cardiotoxicity [1]. Due to its central role in xenobiotic metabolism and abundant cytochrome P450 enzyme system, the liver is especially vulnerable to DOX-induced injury. DOX metabolism generates semiquinone radicals and reactive oxygen species (ROS), leading to oxidative stress, lipid peroxidation, mitochondrial dysfunction, and hepatocellular damage [2,3]. 

Oxidative stress and inflammation are recognized as key mechanisms underlying DOX-induced hepatic injury. Excessive ROS disrupts redox balance, depletes endogenous antioxidant defenses such as superoxide dismutase (SOD), catalase (CAT), and glutathione peroxidase (GPx), and activates pro-inflammatory mediators including nuclear factor kappa B (NF-κB) and tumor necrosis factor alpha (TNF-α). These processes ultimately promote apoptosis and fibrotic remodeling in hepatic tissue [4,5]. Consistently, histopathological alterations such as sinusoidal dilatation, leukocyte infiltration, congestion, and fibrosis have been reported in DOX-treated rodent livers [6]. 

Natural compounds with antioxidant and anti-inflammatory properties have attracted considerable interest as potential protective agents against DOX-induced hepatotoxicity. Hydroxytyrosol (HT), a phenolic compound derived from olive oil, has demonstrated significant hepatoprotective effects in experimental models of oxidative stress and toxic liver injury [7]. HT exerts its protective actions by scavenging ROS, enhancing antioxidant enzyme activities, and modulating inflammatory cytokine expression, thereby preserving hepatic structure and function [8,9].

Recent studies have also emphasized the role of novel molecular mediators in tissue injury and repair. Leucine-rich repeat kinase 2 (LRRK2, dardarin), initially associated with neurodegenerative disorders, has been implicated in inflammatory signaling, autophagy, and oxidative stress regulation [10,11]. Increased LRRK2 expression has been linked to enhanced cytokine production and inflammation in renal and hepatic injury models [12]. Podocalyxin (PODX), a transmembrane sialomucin glycoprotein involved in cell polarity and adhesion, has similarly been associated with fibrosis, cellular dedifferentiation, and pathological remodeling in hepatic tissue when aberrantly expressed [13].

Given these observations, elucidating the involvement of LRRK2 and PODX in DOX-induced hepatotoxicity and determining whether HT modulates their expression may provide important mechanistic insights. Therefore, the present study aimed to investigate the hepatoprotective effects of HT against doxorubicin-induced liver injury in rats, with particular emphasis on the regulation of LRRK2 and PODX expression.

## Materials and methods

**Figure 1:**
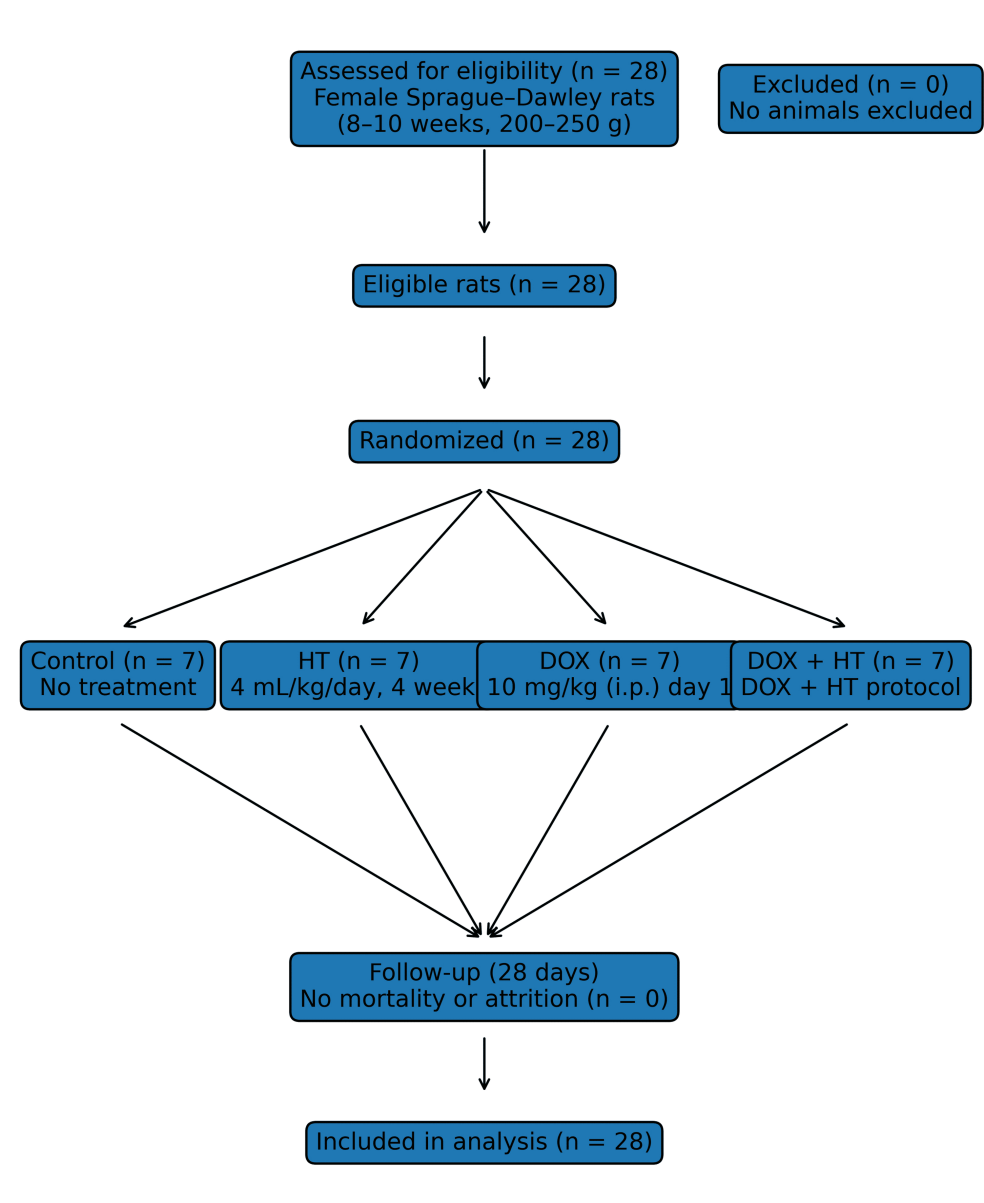
Cohort flow chart of the rat selection and experimental design A total of 28 female Sprague–Dawley rats were assessed for eligibility, and none were excluded. All animals were randomly allocated into four groups (n = 7 per group): Control, HT, DOX, and DOX + HT. No mortality or attrition occurred during the 28-day experimental period, and all animals were included in the final analyses. HT: hydroxytyrosol; DOX: doxorubicin; i.p.: intraperitoneal

This study was conducted in accordance with the ethical standards outlined in the Declaration of Helsinki and approved by the Ethics Committee of Adıyaman University Experimental Animals Department (approval number: 2025/038). All experimental procedures complied with the Guide for the Care and Use of Laboratory Animals [14]. A total of 28 female Sprague-Dawley rats (eight to 10 weeks old, weighing 200-250 g) were obtained from the Adıyaman University Experimental Research Center. All animals were assessed for eligibility, and none were excluded from the study. The rats were housed under controlled environmental conditions (22 ± 2 °C, 12-hour light/dark cycle, 50 ± 10% humidity) with ad libitum access to standard chow and tap water throughout the experimental period. The animals were randomly allocated into four experimental groups (n = 7 per group): (1) Control, (2) HT, (3) DOX, and (4) DOX + HT. The sample size was determined based on previous experimental studies using similar models of DOX-induced hepatotoxicity and comparable histopathological/immunohistochemical outcome measures, while also considering the ethical principle of minimizing animal use. In addition, a post hoc power analysis was performed using the primary immunohistochemical outcome parameters under one-way ANOVA assumptions with an alpha level of 0.05. The analysis demonstrated a statistical power greater than 99%, indicating that the sample size was adequate to detect significant differences among the experimental groups. No mortality or attrition occurred during the study, and all 28 animals completed the experimental protocol and were included in the final analyses. The control group received vehicle solution via oral gavage without active treatment. The control and DOX groups received the same volume of vehicle solution via oral gavage throughout the experimental period to ensure equivalent handling and administration stress among all groups. The liquid form of HT was kindly supplied by Kale Naturel Herbal Products Co. (Balıkesir, Türkiye). Rats in the HT and DOX + HT groups were administered HT orally at a dose of 100 mg/kg/day for four consecutive weeks [15,16]. The DOX group received a single intraperitoneal injection of DOX (10 mg/kg) on the first day of the experiment [17]. Similarly, the DOX + HT group received an intraperitoneal injection of DOX (10 mg/kg) on day 1, followed by oral administration of HT (100 mg/kg/day) for four weeks. At the end of the 28-day experimental period, all animals were anesthetized by intraperitoneal injection of ketamine (75 mg/kg) and xylazine (10 mg/kg). Blood samples were obtained via cardiac puncture as part of the terminal sampling procedure; however, serum biochemical analyses of hepatic injury markers were not performed because the study primarily focused on histopathological and immunohistochemical assessment of liver tissue. Liver tissues were subsequently harvested and fixed in 10% neutral buffered formalin for immunohistochemical evaluation. The process of animal selection, randomization, follow-up, and inclusion in the final analysis is summarized in the cohort flow chart (Figure 1).

Histochemical examination

Liver tissue samples were processed according to standard histological procedures and embedded in paraffin blocks [18]. Serial sections of 5 µm thickness were obtained using a rotary microtome. The sections were subsequently stained with hematoxylin and eosin (H&E) to evaluate general tissue architecture, and Masson’s trichrome staining was performed to assess collagen deposition and fibrosis. For the detection of specific protein expressions, immunohistochemical staining protocols were applied to selected sections.

Immunohistochemical examination

Immunohistochemical analysis was performed on paraffin-embedded liver tissue sections in accordance with standard protocols [18]. Serial sections (3 µm thick) were mounted onto tissue microarray slides. Primary antibodies against LRRK2 (orb500678; Biorbyt Ltd., Cambridge, UK) and PODX (XH358726; Thermo Fisher Scientific, Rockford, IL, USA) were applied for antigen detection. Stained sections were visualized and photographed using a Zeiss Axio Scope A1 microscope (Carl Zeiss Microscopy GmbH, Jena, Germany).

Immunoreactivity was semi-quantitatively evaluated by assessing both the distribution and intensity of staining. For each specimen, at least five randomly selected non-overlapping microscopic fields were examined under identical magnification (×400). The distribution of positive staining was graded as follows: 0 for negative staining, 0.1 for <25% of the stained area, 0.4 for 26%-50%, 0.6 for 515-75%, and 0.9 for 76%-100%. Staining intensity was scored on a four-point scale: 0 (no staining), 1 (weak), 2 (moderate), and 3 (strong).

The histoscore for each specimen was calculated using the following formula: Histoscore = D × I, where D represents the distribution coefficient and I denotes the staining intensity score. Accordingly, the histoscore ranged from 0 (no immunoreactivity) to 2.7 (maximum distribution × strongest intensity).

All slides were evaluated independently by two blinded observers to minimize subjective bias. Any discrepancies in scoring were resolved through joint reevaluation and consensus scoring. Scoring procedures were conducted in accordance with previously described methods [18].

Statistical analysis

All statistical analyses were conducted using the IBM SPSS Software, version 22.0 (IBM Corp., Armonk, NY, USA). Data were expressed as mean ± standard deviation (SD). Prior to statistical comparisons, the assumptions of normality and homogeneity of variance were evaluated using the Shapiro-Wilk and Levene tests, respectively. Since the data met the assumptions required for parametric analysis, differences among groups were analyzed using one-way analysis of variance (ANOVA). When significant differences were identified, Tukey’s honestly significant difference (HSD) post hoc test was applied for multiple comparisons. A p-value of less than 0.05 was considered statistically significant.

## Results

Histochemical findings

**Figure 2:**
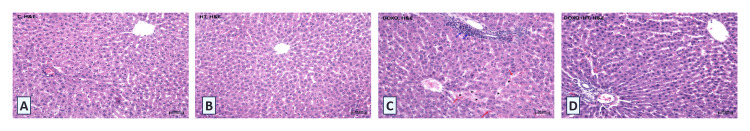
Representative hematoxylin and eosin-stained liver sections showing leukocyte infiltration (blue arrow), congestion (red arrow), and sinusoidal dilation (black triangle); Scale bar: 100 µm; A: Control, B: HT, C: DOX, D: DOX + HT. All images were obtained from the present experimental study using a Zeiss Axio Scope A1 microscope (Carl Zeiss Microscopy GmbH, Jena, Germany). HT: hydroxytyrosol; DOX: doxorubicin

**Figure 3:**
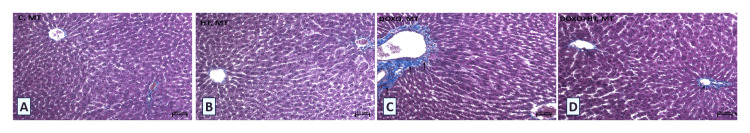
Representative Masson's trichrome-stained liver sections showing fibrosis (black arrow); Scale bar: 100 µm; A: Control, B: HT, C: DOX, D: DOX + HT. All images were obtained from the present experimental study using a Zeiss Axio Scope A1 microscope (Carl Zeiss Microscopy GmbH, Jena, Germany). HT: hydroxytyrosol; DOX: doxorubicin

Histochemical evaluation using hematoxylin-eosin and Masson’s trichrome staining demonstrated preserved hepatic architecture in both the control and HT groups. Fibrosis, leukocyte infiltration, congestion, and sinusoidal dilatation scores were low in these groups, and no statistically significant differences were detected between them (p > 0.05) (Table 1, Figures 2-3).

**Table 1:** istopathologic findings of the liver tissues (hematoxylin & eosin-Masson trichrome) a. p<0.05 compared to control; b. p<0.05 compared to HT; c. p<0.05 compared to DOX. Group comparisons were performed using one-way ANOVA followed by Tukey’s HSD post-hoc test. Data are expressed as mean ± SD, and values of p < 0.05 were accepted as statistically significant. HT: hydroxytyrosol; DOX: doxorubicin; HSD: honestly significant difference

Parameters	Control	HT	DOX	DOX+HT
Fibrosis	1.71±0.49	1.86±0.38	6.57±0.53 ab	2.86±0.69 abc
Leukocyte Infiltration	1.29±0.49	1.14±0.38	6.86±0.38 ab	1.71±0.49 abc
Congestion	2±0.58	1.71±0.49	6.71±0.49 ab	3.71±0.49 abc
Sinusoidal Dilation	2±0.58	1.71±0.49	6.86±0.38 ab	3.57±0.53 abc

In contrast, the DOX-treated group exhibited marked structural alterations. Fibrosis scores increased from 1.71±0.49 in the control group to 6.57±0.53 in the DOX group (p < 0.001). Similarly, leukocyte infiltration (6.86±0.38), congestion (6.71±0.49), and sinusoidal dilatation (6.86±0.38) were significantly elevated compared with both control and HT groups (p < 0.001 for all comparisons). These findings were accompanied by substantial disruption of normal hepatic organization.

Co-administration of HT significantly attenuated DOX-induced histopathological damage. In the DOX+HT group, fibrosis decreased to 2.86±0.69, leukocyte infiltration to 1.71±0.49, congestion to 3.71±0.49, and sinusoidal dilatation to 3.57±0.53, all significantly lower than in the DOX group (p < 0.001). Although minor alterations persisted compared with the control group, overall hepatic architecture was markedly improved.

Immunohistochemical findings

LRRK2 Expression

LRRK2 immunoreactivity differed significantly among groups (Table 2). The control and HT groups exhibited low baseline expression levels (0.07±0.03 and 0.09±0.02, respectively), with no significant difference between them (p > 0.05).

**Table 2:** Immunohistochemical findings for dardarin in the liver tissues a. p<0.05 compared to control; b. p<0.05 compared to HT; c. p<0.05 compared to DOX. Group comparisons were performed using one-way ANOVA followed by Tukey’s HSD post-hoc test. Data are expressed as mean ± SD, and values of p < 0.05 were accepted as statistically significant. HT: hydroxytyrosol; DOX: doxorubicin; HSD: honestly significant difference

Groups	Control	HT	DOX	DOX +HT
Dardarin	0.07±0.03	0.09±0.02	0.29±0.08 ab	0.09±0.02 c

**Figure 4:**
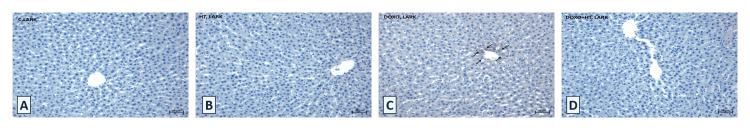
Representative immunohistochemical staining for dardarin in liver tissues (black arrow); Scale bar: 100 µm; A: Control, B: HT, C: DOX, D: DOX + HT. All images were obtained from the present experimental study using a Zeiss Axio Scope A1 microscope (Carl Zeiss Microscopy GmbH, Jena, Germany). HT: hydroxytyrosol; DOX: doxorubicin; HSD: honestly significant difference

The DOX group demonstrated a pronounced increase in LRRK2 expression (0.29±0.08), which was significantly higher than both control and HT groups (p < 0.001). In contrast, the DOX+HT group showed a marked reduction in LRRK2 levels (0.09±0.02) compared with DOX alone (p < 0.001), approaching values observed in the control group. Representative images are shown in Figure 4.

PODX Expression

PODX expression followed a similar pattern (Table 3). Baseline immunoreactivity was minimal in the control (0.19±0.09) and HT (0.21±0.09) groups, without significant intergroup differences (p > 0.05).

**Table 3:** Immunohistochemical findings for PODX in the liver tissues a. p<0.05 compared to control; b. p<0.05 compared to HT; c. p<0.05 compared to DOX. Group comparisons were performed using one-way ANOVA followed by Tukey’s HSD post-hoc test. Data are expressed as mean ± SD, and values of p < 0.05 were accepted as statistically significant. HT: hydroxytyrosol; DOX: doxorubicin; PODX: podocalyxin; HSD: honestly significant difference

Groups	Control	HT	DOX	DOX +HT
PODX	0.19±0.09	0.21±0.09	1.54±0.32 ab	0.66±0.17 abc

**Figure 5:**
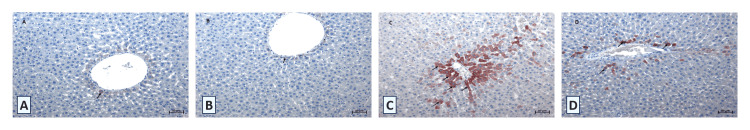
Representative immunohistochemical staining for PODX in liver tissues (black arrow); Scale bar: 100 µm; A: Control, B: HT, C: DOX; D: DOX + HT. All images were obtained from the present experimental study using a Zeiss Axio Scope A1 microscope (Carl Zeiss Microscopy GmbH, Jena, Germany). HT: hydroxytyrosol; DOX: doxorubicin; HSD: honestly significant difference

A substantial increase in PODX expression was observed in the DOX group (1.54±0.32), significantly exceeding both control and HT groups (p<0.001). Co-treatment with HT significantly reduced PODX levels to 0.66±0.17 compared with DOX alone (p < 0.001), although values remained modestly elevated relative to the control group. Representative histological sections are presented in Figure 5.

Overall, the findings demonstrate that DOX administration resulted in significant structural liver damage and marked upregulation of LRRK2 and PODX expression. HT treatment alone did not alter baseline hepatic morphology or protein expression levels. However, co-administration of HT with DOX significantly mitigated histopathological injury and reduced immunohistochemical expression of both markers compared with DOX treatment alone. These results consistently indicate a protective effect of HT against DOX-induced hepatic alterations.

## Discussion

DOX is an effective chemotherapeutic agent whose clinical use is limited by dose-dependent hepatotoxicity. DOX-induced liver injury is mediated by multiple mechanisms, including excessive ROS generation, mitochondrial dysfunction, lipid peroxidation, inflammation, and apoptosis, ultimately leading to hepatocellular degeneration and fibrosis [19]. Oxidative imbalance further activates inflammatory pathways involving TNF-α, interleukin-6 (IL-6), and NF-κB signaling, thereby exacerbating hepatic tissue damage.

HT, a major phenolic compound of olive oil, has been extensively documented for its antioxidant, anti-inflammatory, and cytoprotective properties [20]. Experimental studies indicate that HT effectively scavenges free radicals, restores redox homeostasis, and suppresses pro-inflammatory cytokine production, conferring protection against oxidative damage in various tissues [16].

In the present study, DOX administration induced marked histopathological alterations, including hepatic fibrosis, inflammatory cell infiltration, and sinusoidal dilatation, consistent with previous reports describing oxidative and inflammatory liver injury following DOX exposure. Notably, co-administration of HT significantly ameliorated these pathological changes, indicating a robust hepatoprotective effect of HT against DOX-induced liver damage.

Beyond histopathological injury, DOX treatment was associated with increased expression of LRRK2 and PODX. Importantly, HT administration markedly reduced the expression of both molecules, suggesting their involvement in HT-mediated hepatoprotection. LRRK2 has been linked to inflammatory signaling, mitochondrial dysfunction, and cellular stress responses, while PODX plays a role in cytoskeletal organization and cell adhesion; dysregulation of both proteins has been associated with tissue remodeling and inflammation [21-23]. These findings suggest that the reduced expression of LRRK2/dardarin and PODX following HT treatment may contribute to the hepatoprotective effects of HT; however, further molecular and functional studies are required to clarify the underlying mechanisms.

Our findings are in agreement with previous studies demonstrating that HT mitigates DOX-induced oxidative stress and tissue injury by suppressing lipid peroxidation and enhancing endogenous antioxidant defenses, including SOD, CAT, and GPx [24,25]. Additionally, HT has been shown to inhibit NF-κB activation and reduce pro-inflammatory mediators such as TNF-α and Interleukin-1 beta (IL-1β), thereby attenuating hepatocellular apoptosis and necroinflammatory damage [20].

Taken together, these results indicate that HT exerts hepatoprotective effects not only through direct antioxidant activity but also by modulating molecular pathways associated with oxidative stress, inflammation, and fibrosis. The observed regulation of LRRK2 and PODX highlights new potential molecular targets in the prevention of DOX-induced hepatotoxicity and supports the therapeutic potential of HT as an adjunctive agent.

Several limitations should be acknowledged. The relatively small sample size and use of only female rats may limit generalizability. In addition, molecular techniques such as Western blotting or reverse transcription quantitative polymerase chain reaction (RT-qPCR) were not employed to quantitatively confirm LRRK2 and PODX expression changes. Finally, the study focused on short-term effects; thus, long-term investigations and mechanistic studies involving targeted modulation of LRRK2 and PODX are warranted to further elucidate HT-mediated hepatoprotection.

## Conclusions

In conclusion, the present findings suggest that HT may exert a protective influence in the context of drug-induced hepatic injury. The study highlights the potential relevance of naturally occurring phenolic compounds in maintaining hepatic homeostasis during exposure to cytotoxic agents. While the observed protective tendencies provide a rationale for continued investigation, additional experimental and translational research is required to clarify the underlying biological mechanisms and to determine whether these effects can be translated into clinical benefit.
